# Anatomical Risk Factors for Technical Failure of the Perclose Vascular Closure Device: A Single-Center Retrospective Study

**DOI:** 10.3400/avd.oa.26-00118

**Published:** 2026-10-27

**Authors:** Hirofumi Jinno, Shinsuke Kikuchi, Tsutomu Doita, Izumi Fukii, Kyokei Fuchizawa, Takayuki Uramoto, Seima Ohira, Keisuke Kamada, Naoya Kuriyama, Nobuyoshi Azuma

**Affiliations:** 1 Department of Vascular Surgery, Asahikawa Medical University, Asahikawa, Hokkaido, Japan; 2 Department of Cardiovascular Surgery, Osaka University, Suita, Osaka, Japan; 3 Department of Chronic Limb-Threatening Ischemia Research, Asahikawa Medical University, Asahikawa, Hokkaido, Japan

**Keywords:** Perclose, vascular closure device, endovascular aneurysm repair, common femoral artery, calcification

## Abstract

**Objectives:**

Percutaneous large-bore femoral closure with the Perclose device is widely used, but technical failure remains clinically important. We identified anatomical risk factors for technical failure.

**Methods:**

We retrospectively analyzed endovascular aortic repair and endovascular therapy procedures using the Perclose device at the common femoral artery (CFA) between April 2020 and March 2025. Preoperative computed tomography (CT) measured CFA diameter, puncture depth, and a calcification score (0–4). Technical failure was defined as failure to achieve hemostasis with the intended deployment. Independent predictors were identified using patient-clustered generalized estimating equations.

**Results:**

Among 300 patients (485 limbs), technical failure occurred in 52 limbs (10.7%), of which 75.0% required surgical repair. Independent predictors were a higher calcification score (odds ratio [OR] 2.13 per 1-point increase; 95% confidence interval [CI] 1.37–3.31), an emergency procedure (OR 3.77; 1.55–9.14), a smaller CFA diameter (OR 1.16 per 1-mm decrease; 1.01–1.32), and a shallower puncture depth (OR 1.22 per 5-mm decrease; 1.01–1.47); the area under the curve was 0.72.

**Conclusions:**

Technical failure was driven by unfavorable CFA anatomy—heavy calcification, a small diameter, and a shallow puncture depth—and by emergency procedures. Preprocedural imaging assessment is essential; alternative closure or surgical cutdown should be considered in unfavorable or emergent cases.

## Introduction

Percutaneous femoral access has become central to contemporary endovascular therapy. Large-caliber sheaths for procedures such as endovascular aortic repair (EVAR) traditionally required surgical groin cutdown, but suture-mediated vascular closure devices (VCDs) now permit fully percutaneous access and closure, shortening procedures, reducing wound-related morbidity, and facilitating early mobilization.^1,2)^ The Perclose suture-mediated system (Abbott Vascular, Santa Clara, CA, USA) is among the most widely used devices for large-bore access because of its efficiency and its ability to “pre-close” the arteriotomy before sheath upsizing.^3)^

Despite these advantages, access-site complications—bleeding, pseudoaneurysm, arterial dissection, and stenosis or occlusion—remain clinically relevant, and a nontrivial proportion of deployments fail to achieve the intended hemostasis; reported failure modes include device detachment, failed deployment, and entrapment.^4–6)^ When closure fails, the consequences are frequently serious, ranging from prolonged manual compression to emergency surgical repair of the femoral artery. Successful deployment is understood to depend on common femoral artery (CFA) anatomy, sheath size, puncture location, and operator factors, yet the relative contribution of these variables in routine, unselected practice is incompletely defined, and much of the available evidence derives from cohorts dominated by transcatheter aortic-valve or coronary procedures rather than the mixed EVAR and endovascular therapy (EVT) population encountered in vascular surgical practice.

Two methodological aspects have been handled inconsistently in prior work. First, anatomical risk factors are commonly dichotomized at data-derived thresholds that are unstable and poorly transportable between cohorts. Second, bilateral procedures—common in aortic work—generate 2 correlated observations per patient that are frequently analyzed as if they were independent, which can distort inference. We therefore performed a single-center study of consecutive Perclose closures at the CFA, using preprocedural computed tomography (CT) to characterize the access site and a patient-clustered model that treats the key anatomical variables as continuous, aiming to identify robust risk factors for technical failure and to inform safer patient selection and procedural planning.

## Materials and Methods

### Study design, setting, and ethics

This was a single-center, retrospective observational study conducted at the Department of Vascular Surgery, Asahikawa Medical University Hospital, a tertiary referral center. We reviewed consecutive patients who underwent EVAR or EVT via the CFA in which the Perclose ProGlide or ProStyle suture-mediated closure device was used for arteriotomy closure between April 2020 and March 2025. The protocol was approved by the Institutional Review Board of Asahikawa Medical University (approval number: 25171). Given the retrospective, non-interventional design, the requirement for written informed consent was waived, and patients were offered the opportunity to decline participation through a public opt-out notice, in accordance with national ethical guidelines. The study was designed, conducted, and reported in accordance with the STROBE statement for observational studies.^7)^ Clinical, procedural, and imaging data were extracted from the electronic medical record and the picture-archiving and communication system.

### Participants

All consecutive procedures during the study period that used the Perclose device for CFA access were screened for eligibility. Because hemostasis is a limb-level outcome, each treated limb was analyzed separately, and bilateral procedures contributed 2 observations. Limbs were excluded if the Perclose device was used outside its intended indication (e.g., a superficial femoral or antegrade puncture), if essential preprocedural CT measurements or the closure outcome were missing or unavailable, or if the patient died in the immediate perioperative period before the access site could be evaluated. After excluding 4 patients (5 limbs) for these reasons, 300 patients (485 limbs) were analyzed; the flow of participants is summarized in **Supplementary Fig. S1**. Procedures were performed by 20 vascular surgeons of varying experience.

### Data collection

For each limb, we recorded operator-related variables (cumulative prior Perclose use and postgraduate years), patient characteristics (age, sex, body mass index, hypertension, diabetes, dyslipidemia, smoking, hemodialysis, estimated glomerular filtration rate, active malignancy, prior inguinal intervention, and antithrombotic and steroid therapy), the preprocedural ankle–brachial index, procedural variables (elective vs emergency status, sheath size, number of devices, closure technique, and device generation), and the anatomical measurements described below. The outcomes were the success or failure of Perclose closure and any access-related complication.

### Anatomical evaluation

Preprocedural contrast-enhanced CT was used to characterize the intended access site. Because the exact puncture point could not always be identified retrospectively, the ideal puncture site was defined a priori as the most superficial segment of the CFA with the least circumferential calcification, reflecting the site an operator would preferentially target. On this segment, we measured, on axial images, the CFA diameter (the maximum luminal diameter, excluding calcified regions), the puncture depth (the shortest perpendicular distance from the skin surface to the anterior arterial wall), and the CFA length (from the inguinal ligament to the femoral bifurcation). Access-site calcification was graded by its circumferential extent on a 5-point scale (0, none; 1, <90 degrees; 2, 90–180 degrees; 3, 180–270 degrees; 4, >270 degrees).

### Procedural technique

All arterial punctures were performed under ultrasound guidance. The closure strategy was determined by sheath size: a pre-close technique was used for sheaths of 8 Fr or larger, and a post-close technique was used for sheaths of 7 Fr or smaller; a single device was deployed for sheaths up to 12 Fr, and 2 devices for sheaths of 13 Fr or larger. After the sutures were tied, manual compression was applied, and hemostasis was confirmed by ultrasound before a compression dressing was placed and the patient returned to the ward. The decision to use the Perclose device, and the number of devices, was at the discretion of the attending operator.

### Outcomes and definitions

The primary outcome was technical failure, defined as failure to achieve complete hemostasis with the initially intended Perclose deployment, requiring any additional measure (prolonged manual compression, surgical cutdown, or endovascular treatment). Procedures achieving hemostasis with the intended deployment were classified as technical successes. Access-related complications comprised hematoma, pseudoaneurysm, bleeding requiring intervention, arterial dissection, and arterial stenosis or occlusion, whether they occurred at the time of the procedure or during the in-hospital course. For every failed limb, the mode of failure and the subsequent management (manual compression, thrombus aspiration, surgical suturing, femoral endarterectomy, or arterial reconstruction) were recorded.

### Statistical analysis

Analyses were performed with SPSS 28.0 (IBM, Armonk, NY, USA) and R 4.4 (R Foundation for Statistical Computing, Vienna, Austria), using the geepack package for generalized estimating equations and the pROC package for receiver-operating characteristic analysis; all analyses were additionally re-run in R, which reproduced the original estimates. Continuous variables are presented as mean ± standard deviation, and categorical variables are presented as numbers and percentages; groups were compared with the Mann–Whitney U test and the chi-square or Fisher exact test, as appropriate. Six limbs with physiologically implausible CT measurements (puncture depth >60 mm or CFA diameter >20 mm) were treated as missing and confirmed not to affect the results in sensitivity analyses; together with 4 limbs that had missing model covariates, 10 limbs were omitted from the multivariable model, which was fitted on the remaining 475 limbs (52 events). Because many patients contributed 2 limbs (196 bilateral procedures), the observations were not independent; independent predictors of technical failure were therefore identified using binary logistic regression fitted with generalized estimating equations and an exchangeable working correlation clustered by patient, which yields cluster-robust confidence intervals (CIs).^8)^ Candidate variables were selected on clinical grounds and univariate association (P <0.20). CFA diameter and puncture depth were modeled as continuous variables because data-derived (receiver-operating characteristic) cut-offs proved unstable across sensitivity analyses (for example, dichotomizing calcification at a score of 3 produced an unstable odds ratio [OR] of 8.16 [95% CI 2.49–26.79], which modeling the ordinal score markedly stabilized); the calcification score was modeled across its ordinal range. A Firth-penalized model was used as a sensitivity analysis for the sparse high-calcification stratum,^9)^ and model discrimination was summarized by the area under the receiver-operating characteristic (ROC) curve. In an exploratory analysis, the same patient-clustered model was applied with surgical conversion—failure managed by arterial reconstruction or angioplasty, femoral endarterectomy, or surgical suturing—as the outcome. To interrogate the operator-experience association, we additionally screened all recorded variables for association with both experience and failure, decomposed cumulative experience into within- and between-operator components, and applied cluster-robust adjustment by operator. A 2-sided P <.05 was considered statistically significant.

## Results

### Patient and limb characteristics

Between April 2020 and March 2025, 300 patients (485 limbs) met the eligibility criteria and were analyzed; participant flow is summarized in **Supplementary Fig. S1**. Baseline clinical and procedural characteristics are shown in **Table 1**. Technical failure occurred in 52 of 485 limbs (10.7%). Compared with successful limbs, failed limbs had a smaller CFA diameter (8.5 ± 1.7 vs 9.3 ± 2.2 mm; P = 0.004), a shallower puncture depth (17.9 ± 8.6 vs 21.9 ± 10.3 mm; P = 0.012), a higher calcification score (1.23 ± 0.94 vs 0.78 ± 0.71; P = 0.001), and a higher proportion of severe (≥180°) circumferential calcification (9.6% vs 1.6%; P = 0.005). Emergency procedures were more frequent among failures (19.2% vs 7.9%; P = 0.017). There were no significant differences in the ankle–brachial index, sheath size, number of devices, history of inguinal intervention, atherosclerotic risk factors, or antithrombotic and steroid use. Anterior-wall calcification specifically was uncommon (present in 10 limbs) but was associated with failure: 4 of 10 such limbs (40.0%) failed vs 10.1% of limbs without anterior-wall calcification (P = 0.015). Although failures were somewhat more common with the ProStyle than with the ProGlide generation on univariate comparison (67.3% vs 51.5%; P = 0.039), this difference did not persist after adjustment (below).

**Table 1:** Baseline clinical and procedural characteristics

Variable	Success (n = 433)	Failure (n = 52)	P value
Age (years)	78.6 ± 7.5	80.7 ± 7.7	0.089
Male sex	358 (82.7)	38 (73.1)	0.127
Body mass index (kg/m^2^)	23.0 ± 3.8	22.1 ± 3.0	0.053
Hypertension	337 (77.8)	40 (76.9)	0.861
Diabetes mellitus	103 (23.8)	14 (26.9)	0.609
Dyslipidemia	229 (52.9)	29 (55.8)	0.769
Current smoking	151 (34.9)	15 (28.8)	0.441
Hemodialysis	10 (2.3)	2 (3.8)	0.375
eGFR (mL/min/1.73 m^2^)	56.8 ± 19.1	57.8 ± 23.2	0.782
Single antiplatelet therapy	131 (30.3)	19 (36.5)	0.346
Dual antiplatelet therapy	28 (6.5)	6 (11.5)	0.243
Warfarin	17 (3.9)	2 (3.8)	1.000
DOAC	48 (11.1)	6 (11.5)	0.819
Steroid therapy	29 (6.7)	2 (3.8)	0.561
Preprocedural ABI	0.98 ± 0.26	0.94 ± 0.32	0.254
Emergency procedure	34 (7.9)	10 (19.2)	0.017
Sheath size (Fr)	13.2 ± 4.1	13.8 ± 4.2	0.588
Devices per case	1.59 ± 0.71	1.60 ± 0.60	0.976
ProStyle device (vs ProGlide)	223 (51.5)	35 (67.3)	0.039
Puncture depth (mm)	21.9 ± 10.3	17.9 ± 8.6	0.012
CFA diameter (mm)	9.3 ± 2.2	8.5 ± 1.7	0.004
CFA length (mm)	31.5 ± 11.0	31.6 ± 12.9	0.967
Calcification score (0–4)	0.78 ± 0.71	1.23 ± 0.94	0.001
Calcification score ≥3	7 (1.6)	5 (9.6)	0.005

Values are mean ± SD or n (%).

ABI, ankle–brachial index; CFA, common femoral artery; DOAC, direct oral anticoagulant; eGFR, estimated glomerular filtration rate; SD, standard deviation

### Failure patterns and management

Failure patterns and their management are summarized in **Table 2**. Each limb was assigned a single, mutually exclusive failure pattern. Inadequate hemostasis and knot or suture disruption were the most frequent patterns, each accounting for 36.5% of failures; less common patterns were stenosis, including residual device material (11.5%), insertion difficulty (5.8%), dissection (5.8%), and occlusion (3.8%). Failure was rarely benign: surgical repair was required in 39 limbs (75.0%), comprising arterial reconstruction or angioplasty (40.4%), femoral endarterectomy (21.2%), and surgical suturing (13.5%); manual compression alone sufficed in 19.2%, and 2 limbs were not repaired because of in-hospital death. In an exploratory patient-clustered analysis with surgical conversion (39 limbs) as the outcome, a higher calcification score was the only independent predictor (OR 1.76 per 1-point increase; 95% CI 1.10–2.84; P = 0.019), with nonsignificant trends for emergency status (OR 2.54; P = 0.083) and a shallower puncture depth (P = 0.067) and no association for CFA diameter (**Supplementary Table S1**). Access-related complications were not confined to overt failures—even among limbs classified as technically successful, approximately 2.5% developed an access-site problem during the procedure or hospital stay, underscoring that immediate apparent success does not guarantee an uncomplicated course.

**Table 2:** Patterns and management of technical failure (n = 52 limbs)

Characteristic	n (%)
Failure pattern	
Knot/suture disruption	19 (36.5)
Inadequate hemostasis	19 (36.5)
Stenosis (incl. residual material)	6 (11.5)
Insertion difficulty	3 (5.8)
Dissection	3 (5.8)
Occlusion	2 (3.8)
Management	
Arterial reconstruction/angioplasty	21 (40.4)
Femoral endarterectomy	11 (21.2)
Manual compression	10 (19.2)
Surgical suturing	7 (13.5)
None (in-hospital death)	2 (3.8)
Thrombus aspiration	1 (1.9)

### Independent anatomical predictors

In the patient-clustered multivariable model (475 limbs, 52 events), 4 factors were independently associated with technical failure (**Table 3** and **Fig. 1A**): a higher calcification score (OR 2.13 per 1-point increase; 95% CI 1.37–3.31; P = 0.001), an emergency procedure (OR 3.77; 95% CI 1.55–9.14; P = 0.003), a smaller CFA diameter (OR 1.16 per 1-mm decrease; 95% CI 1.01–1.32; P = 0.030), and a shallower puncture depth (OR 1.22 per 5-mm decrease; 95% CI 1.01–1.47; P = 0.037). Modeling calcification continuously across its 0–4 range used the full ordinal gradient and yielded a far more stable estimate than a sparse high-grade dichotomy (per-point OR 2.13 vs OR 8.16, 95% CI 2.49–26.79, for a score ≥3); the effect was concentrated at scores ≥2 (circumferential calcium ≥180°). The model discriminated moderately well (area under the curve [AUC] 0.72; 95% CI 0.64–0.79; **Supplementary Fig. S2**), and the predicted probability of failure rose steadily as CFA diameter and puncture depth decreased (**Fig. 1B** and **1C**). Other candidate variables—including body mass index, ankle–brachial index, CFA length, comorbidities, sheath size, device generation, and operator seniority—were tested but were not independently associated with failure.

**Table 3:** Multivariable analysis of independent predictors of technical failure

Variable	Odds ratio	95% CI	P value
Calcification score (per 1-point increase)	2.13	1.37–3.31	0.001
Emergency procedure	3.77	1.55–9.14	0.003
CFA diameter (per 1-mm decrease)	1.16	1.01–1.32	0.030
Puncture depth (per 5-mm decrease)	1.22	1.01–1.47	0.037

Patient-clustered logistic regression (generalized estimating equations); 475 limbs, 52 events; model AUC 0.72. Diameter, depth, and the calcification score were modeled continuously (see Materials and Methods). Operator experience was not an independent predictor and is not shown (see text).

AUC, area under the curve; CFA, common femoral artery; CI, confidence interval

**Fig. 1:**
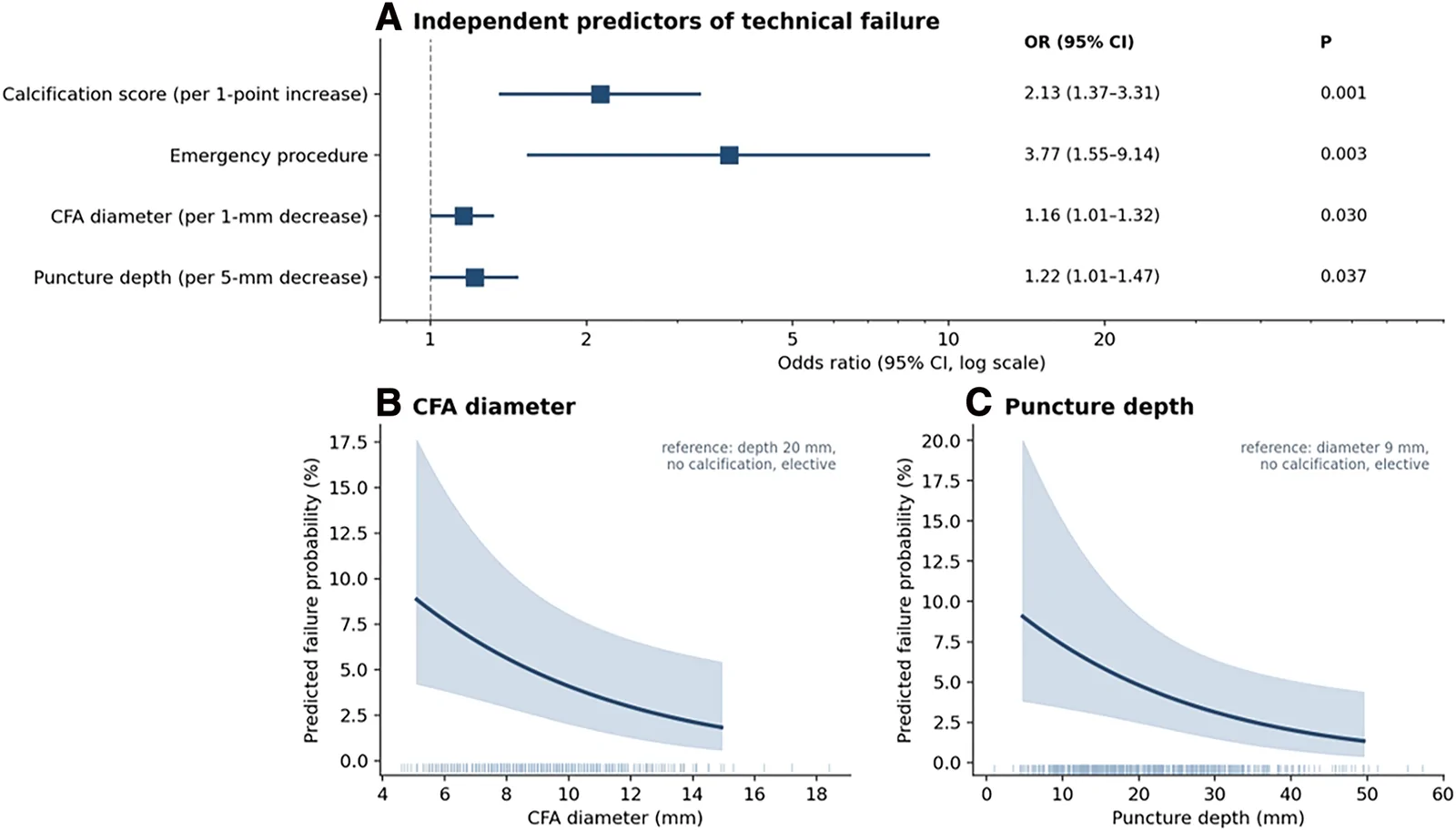
Anatomical determinants of Perclose technical failure. (**A**) Independent predictors from the multivariable logistic-regression model fitted with generalized estimating equations to account for clustering of limbs within patients (475 limbs, 52 events; area under the curve, 0.72): squares are odds ratios, and horizontal lines are 95% confidence intervals. The calcification score is expressed per 1-point increase (range 0–4); emergency procedure is binary; and CFA diameter and puncture depth are continuous, expressed per 1-mm and per 5-mm decrease, respectively, so that the odds increase as the vessel becomes smaller or more superficial; the dashed line marks the null value (odds ratio 1). (**B**, **C**) Predicted probability of technical failure across the observed range of CFA diameter (**B**) and puncture depth (**C**). Solid lines are model-predicted probabilities with the other covariates fixed at reference values (no calcification, elective procedure, and—for each panel—the other anatomical variable at its reference value: CFA diameter, 9 mm; puncture depth, 20 mm); shaded areas are 95% confidence bands, and tick marks along the horizontal axis show the distribution of observed values. CFA, common femoral artery; CI, confidence interval

### Operator experience and case-mix

Counterintuitively, procedures performed by more experienced operators (>10 prior Perclose cases) showed a higher unadjusted failure rate than those performed by early-experience operators, and this direction persisted after adjustment for anatomy. The pattern reflected case-mix rather than skill: experienced operators handled more emergent procedures (10.6% vs 2.3%), more large-bore access (sheath ≥16 Fr, 54.3% vs 40.2%), and more multi-device closures (58.3% vs 46.0%), whereas access-site calcification did not differ between the groups (**Fig. 2A** and **2B**). Cumulative adjustment for these complexity factors, device generation, and study year attenuated the excess (OR from 6.5 to 4.3) but did not abolish it, consistent with residual confounding by unmeasured case difficulty. Device generation (ProStyle vs ProGlide) was not independently associated with failure after adjustment for anatomy (OR 1.70; 95% CI 0.86–3.37; P = 0.13). No learning curve was demonstrable by any pre-specified analysis (**Fig. 2C** and **2D**): failure rates were similar below and above a literature-based 40-case proficiency threshold (10.1% vs 11.4%), quartiles of experience showed no monotonic gradient, a restricted cubic spline was flat, and cumulative experience discriminated failure poorly (AUC 0.54). Operator experience was therefore treated as a descriptive, confounded observation and excluded from the outcome model.

**Fig. 2:**
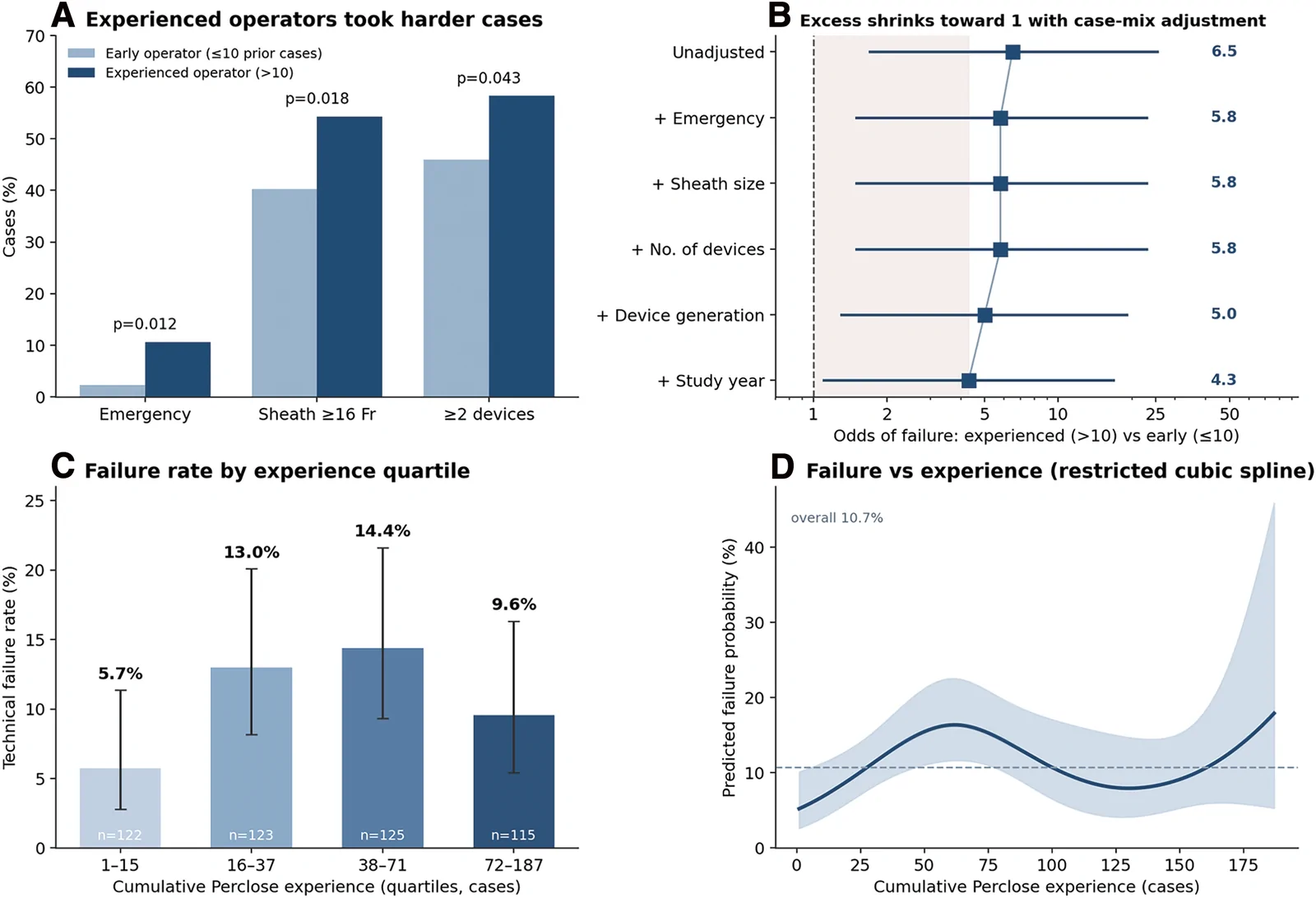
Operator experience reflects case-mix, not a learning curve. (**A**) Proportion of procedures with high-complexity features—emergency status, large-bore access (sheath ≥16 Fr), and multi-device closure (≥2 devices)—among operators with ≤10 vs >10 prior Perclose cases; more experienced operators handled more complex access (Fisher exact P values shown). (**B**) Odds of technical failure for experienced (>10) vs early-experience (≤10) operators, with cumulative adjustment for measured case-mix; each row adds 1 variable to the model above it (emergency status, sheath size, number of devices, device generation, and study year). Squares are odds ratios, and horizontal lines are 95% confidence intervals; the dashed line marks the null value. The apparent excess risk falls from 6.5 (unadjusted) to 4.3 (fully adjusted) but does not reach 1, consistent with residual confounding by unmeasured case difficulty rather than a learning curve. (**C**) Technical-failure rate by quartile of cumulative Perclose experience (error bars, 95% confidence intervals); the rate does not decline with experience, and the confidence intervals overlap. (**D**) Restricted cubic spline of the predicted failure probability against cumulative experience (shaded area, 95% confidence band; dashed line, overall failure rate, 10.7%); the curve is flat and non-monotonic. Cumulative experience discriminated failure poorly (area under the curve, 0.54), and failure rates were similar below and above the literature-based 40-case proficiency threshold (10.1% vs 11.4%).

## Discussion

In this patient-clustered analysis of 485 limbs, technical failure of Perclose closure was driven by unfavorable CFA anatomy—heavy calcification, a small diameter, and a shallow puncture depth—and by emergency procedures, with the risk of failure rising continuously as the vessel became smaller or more superficial. The apparent effect of operator experience was explained largely by case selection rather than by a learning curve. Because every anatomical predictor is measurable on the preprocedural CT that these patients already undergo, the findings translate directly into access planning.

### Anatomical factors

A higher calcification score was the strongest anatomical predictor (OR 2.13 per 1-point increase), with the effect concentrated at scores ≥2 (circumferential calcium ≥180°). Circumferential calcium can deflect the needle, prevent the device foot from apposing the arterial wall, and impede knot advancement, leaving a residual arteriotomy gap; these mechanisms are consistent with the observed predominance of knot or suture disruption and inadequate hemostasis among the failure patterns and with prior reports identifying anterior-wall calcification as a determinant of failure.^4,10,11)^ Modeling calcification continuously rather than as a rare high-grade dichotomy markedly improved the stability of the estimate and avoided reliance on a sparse subgroup. A smaller CFA diameter (OR 1.16 per 1-mm decrease) offers a narrower target for suture capture and less tissue over which to distribute closure tension,^10,12)^ while an unusually shallow artery (OR 1.22 per 5-mm decrease) shortens the subcutaneous suture tract and may predispose to knot entrapment near the inguinal ligament.^13)^ Notably, in a large-bore (>16 Fr) cohort, a greater puncture depth and a higher body mass index predicted failure,^4)^ the opposite of the depth relationship we observed; this contrast underscores that anatomical thresholds are cohort-dependent and argues against transporting fixed cut-offs across populations. In our lean cohort (mean body-mass index 22.9 kg/m^2^) the failure rate fell monotonically across depth quartiles, from 14.8% at the shallowest to 7.4% at the deepest, placing these patients on the shallow end of the depth spectrum, where a short subcutaneous tract with knot entrapment near the inguinal ligament—rather than difficult deep-suture management in obese arteries—is the likely mechanism. Consistent with this, an extremely superficial CFA has recently been reported as an independent cause of Perclose failure: Haba et al. described bilateral closure failure in an emaciated patient (body mass index 15.2) whose common femoral arteries lay only 3 mm beneath the skin—the suture tracts traversed the skin and subcutaneous tissue rather than reaching the arterial wall—and recommended primary surgical cutdown when CFA depth is below 8 mm^14)^; our population-level analysis of 485 limbs quantifies and generalizes that case-level observation. A low body mass index was associated with failure on univariate analysis but not independently, and it correlated strongly with puncture depth (Spearman ρ = 0.68), indicating that its apparent effect operates through a more superficial CFA rather than through body habitus per se. Because these relationships are continuous, we deliberately modeled diameter and depth as continuous variables rather than imposing data-derived cut-offs, which proved unstable across sensitivity analyses; the predicted failure probability rose smoothly as the vessel became smaller or more superficial (**Fig. 1B** and **1C**), providing a graded framework for risk assessment rather than an artificial threshold. Emergency status carried an independently elevated risk (OR 3.77), plausibly reflecting hemodynamic instability, coagulopathy, and hurried, unplanned access^15)^; notably, this association was masked in our earlier modeling by a confounded operator-experience term and became apparent only once that term was removed.

### Operator experience: A case-mix effect, not a learning curve

When analyzed correctly, greater cumulative experience was associated with higher—not lower—failure, the opposite of a learning-curve hypothesis. The most parsimonious explanation is confounding by case assignment: more experienced operators were preferentially referred emergent, large-bore, and multi-device access, and adjustment for these factors, device generation, and study year attenuated but did not eliminate the association (**Fig. 2A** and **2B**), consistent with residual confounding by unmeasured case difficulty. These operator-level analyses are further limited by repeated procedures within a small number of operators (20 operators, 2–95 procedures each), which entangles within- and between-operator effects; on decomposition, neither component was independently associated with failure, and experience discriminated failure poorly (AUC 0.54). Importantly, no learning curve was demonstrable using pre-specified literature thresholds rather than a data-derived cut-off:^16,17)^ failure rates were similar for ≤40 vs >40 prior cases (10.1% vs 11.4%), quartiles showed no monotonic gradient, and a spline of failure against experience was flat (**Fig. 2D**). The very low failure rate confined to each operator’s earliest cases—rather than a genuine proficiency threshold—produced the spurious appearance of a ≤10-case advantage. We therefore report operator experience as a descriptive, confounded observation and excluded it from the outcome model. Beyond this cohort, the episode is a useful cautionary example: operator-volume associations derived from observational data are highly susceptible to case-selection bias, and an uncorrected analysis can reverse the apparent direction of effect. Consistent with the absence of a demonstrable learning curve, the institutional failure rate did not fall but rose between the earlier and later halves of the study (fiscal years 2020–2022, 7.0%, vs 2023–2025, 15.6%; P = 0.003), which we attribute to more complete ascertainment of subtle failures and to increasingly complex and emergent referrals rather than to changing technique; a genuine within-operator learning curve—recognized for this device chiefly in procedure- and fluoroscopy-time metrics rather than in the closure-failure rate—could not be assessed, because the operators’ complete chronological case sequences, including cases performed at other institutions, were unavailable.

### Clinical implications

Failure was consequential: 3-quarters of failed limbs required surgical repair, including femoral endarterectomy (21.2%) and arterial reconstruction or angioplasty (40.4%). Our overall failure rate (10.7%) and this high proportion requiring surgical repair should be interpreted in the context of a deliberately strict failure definition—any deviation from hemostasis achieved with the single intended deployment—applied to a consecutive, real-world tertiary-referral and teaching cohort that included emergent and heavily calcified access; on a comparable device-related-complication endpoint, the rate is essentially identical to that in real-world percutaneous-EVAR series (approximately 10%), and the high surgical-repair proportion reflects definitive vascular-surgical management of failures—particularly calcified ones, which our analysis identified as the principal driver of surgical conversion. In patients with heavy calcification, a small CFA diameter, or a shallow puncture depth—particularly when several of these coexist—alternative closure strategies or primary surgical cutdown should be considered before device deployment, and when a device is used, access should be planned with skin-incision placement and proximal control that anticipate conversion.^18,19)^ The graded probability estimates in **Fig. 1B** and **1C** can support this discussion at the bedside without recourse to a rigid cut-off. Ultrasound-guided puncture and echo-confirmed deployment may improve accuracy:^20,21)^ in our cohort, 2 experienced operators routinely used a long-axis ultrasound view of the CFA to confirm that the device needle engaged the anterior arterial wall before deployment. Although this practice was exploratory and limited to 27 procedures by these 2 operators (none with calcification ≥3), the failure rate in echo-guided cases was numerically lower (3.7% vs 11.1%; P = 0.34), consistent with reports that echo-guided ProGlide deployment may improve accuracy and warranting prospective evaluation.^20)^ Finally, because access-site problems occasionally arose even after apparently successful closure, we favor routine completion imaging and a low threshold for post-procedural surveillance in anatomically high-risk limbs.

### Limitations

This study has several limitations. First, the actual puncture site could not always be identified retrospectively, so anatomy was characterized at the pre-defined ideal site on CT rather than at the true entry point. Second, intraluminal factors such as intimal thickening, mural thrombus, and focal stenosis were not fully assessed. Third, this was a retrospective, single-center study, which limits generalizability and precludes control of unmeasured confounders. Fourth, a small number of physiologically implausible CT measurements were excluded; the results were unchanged in sensitivity analyses. Fifth, the observed failure rate rose over the study period, which may partly reflect more complete ascertainment of failures in recent years rather than a true temporal trend. Sixth, operator experience was confounded by case assignment and was not analyzed as a causal factor. Finally, the anatomical risk gradient, although internally robust, requires external validation before it can be applied prospectively.

## Conclusion

Technical failure of Perclose closure was determined chiefly by CFA anatomy—severe calcification, a small diameter, and a shallow puncture depth—and by emergency procedures, with failure risk rising continuously as the vessel became smaller or more superficial. Careful preprocedural imaging assessment and appropriate case selection are essential, and alternative closure or primary surgical cutdown should be considered in anatomically unfavorable or emergent cases. The apparent association with operator experience reflected case selection rather than a learning curve and should not be interpreted causally.

## Supplementary Materials

Supplementary Fig. S1 Flow of participants through the study (STROBE). Of 304 patients (490 limbs) undergoing Perclose closure of femoral access during the study period, 4 patients (5 limbs) were excluded—a ruptured case with perioperative death before access-site assessment (2 limbs), off-label use (1 superficial femoral and 1 antegrade puncture; 2 limbs), and 1 case with unclear surgical records (1 limb)—leaving a study population of 300 patients (485 limbs). Technical success was achieved in 433 limbs (89.3%), and technical failure occurred in 52 limbs (10.7%).

Supplementary Fig. S2 Receiver-operating characteristic curve of the multivariable model for technical failure. The area under the curve was 0.72 (95% CI 0.64–0.79), indicating moderate discrimination; the dashed diagonal represents chance.

Supplementary Table S1 Univariable and multivariable analyses of predictors of surgical conversion (arterial reconstruction/angioplasty, femoral endarterectomy, or surgical suturing; 39 of 485 limbs), estimated by logistic regression with generalized estimating equations clustered by patient. A higher calcification score was the only independent predictor.
