# Supplementary material for: Anatomical Risk Factors for Technical Failure of the Perclose Vascular Closure Device: A Single-Center Retrospective Study
Source: Ann Vasc Dis. 2026 Oct 27;19(1):26-00118. doi: 10.3400/avd.oa.26-00118 (PMC13724158; doi:10.3400/avd.oa.26-00118)
Supplement: Supplementary file 1 [file avd-19-1-26-00118-s001.pdf]

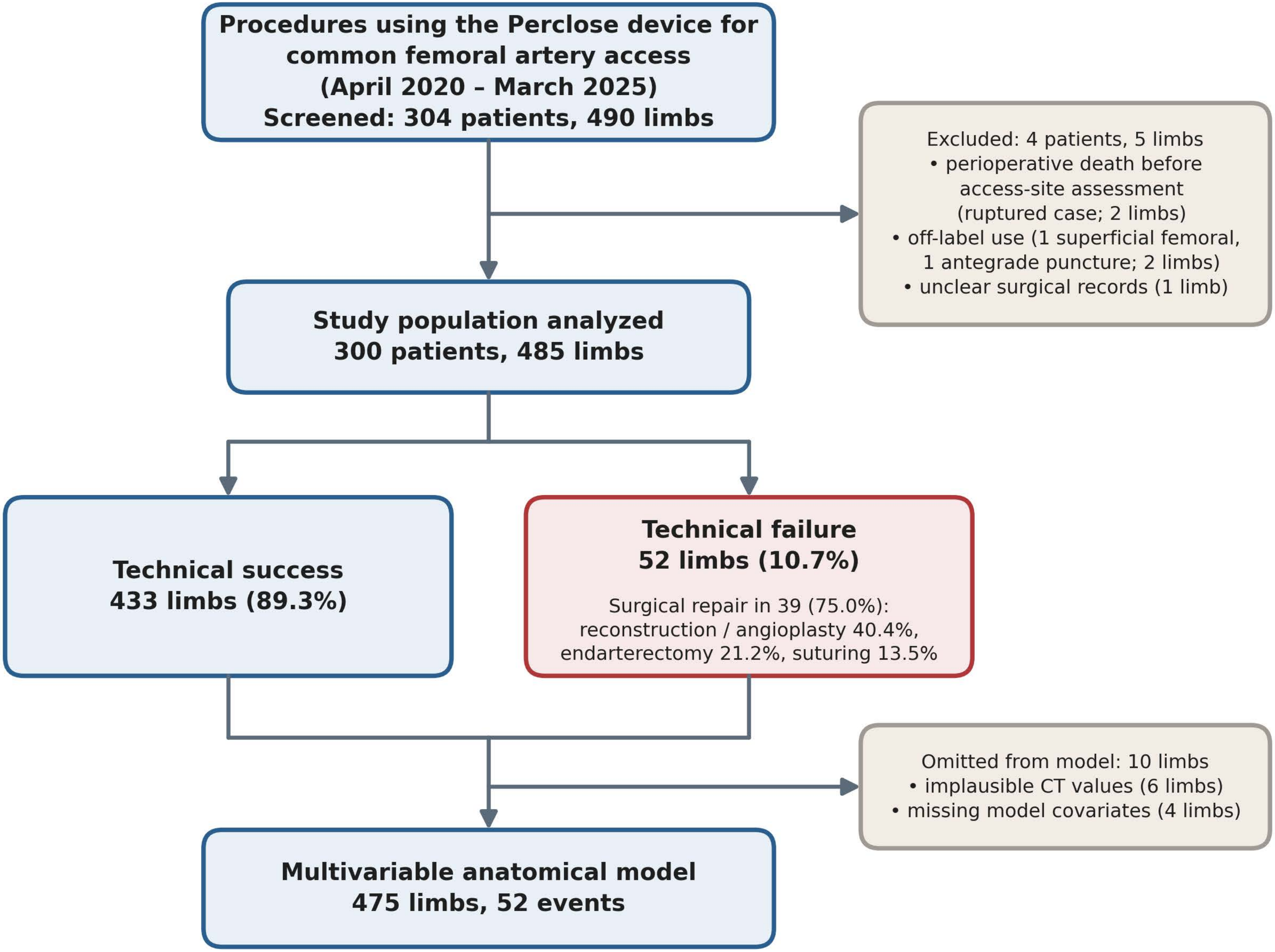
Procedures using the Perclose device for common femoral artery access (April 2020 – March 2025)
Screened: 304 patients, 490 limbs
Excluded: 4 patients, 5 limbs
• perioperative death before access-site assessment (ruptured case; 2 limbs)
• off-label use (1 superficial femoral, 1 antegrade puncture; 2 limbs)
• unclear surgical records (1 limb)
Study population analyzed
300 patients, 485 limbs
Technical success
433 limbs (89.3%)
Technical failure
52 limbs (10.7%)
Surgical repair in 39 (75.0%):
reconstruction / angioplasty 40.4%,
endarterectomy 21.2%, suturing 13.5%
Omitted from model: 10 limbs
• implausible CT values (6 limbs)
• missing model covariates (4 limbs)
Multivariable anatomical model
475 limbs, 52 events

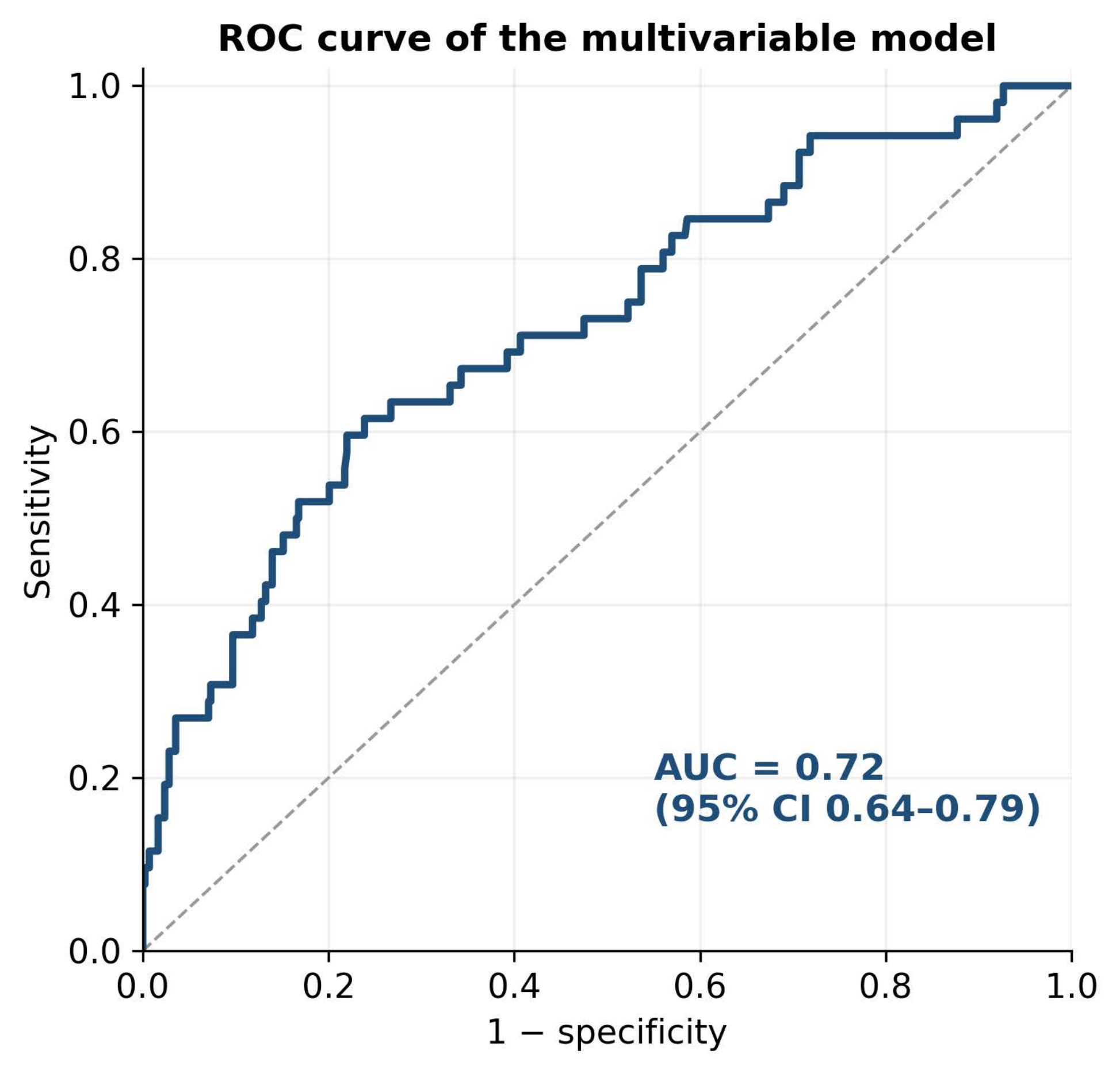

ROC curve of the multivariable model
Sensitivity
1 − specificity
0.0
0.2
0.4
0.6
0.8
1.0
AUC = 0.72
(95% CI 0.64–0.79)
