# Supplementary material for: Anatomical Risk Factors for Technical Failure of the Perclose Vascular Closure Device: A Single-Center Retrospective Study
Source: Ann Vasc Dis. 2026 Oct 27;19(1):26-00118. doi: 10.3400/avd.oa.26-00118 (PMC13724158; doi:10.3400/avd.oa.26-00118)
Supplement: Supplementary file 2 [file avd-19-1-26-00118-s002.pdf]

Supplementary Table S1 Univariable and multivariable analysis of predictors of surgical conversion

Variable	Univariable OR (95% CI)	P	Multivariable OR (95% CI)	P
Calcification score (per 1-point increase)	1.78 (1.11–2.84)	0.016	1.76 (1.10–2.84)	0.019
Emergency procedure	2.37 (0.85–6.54)	0.097	2.54 (0.88–7.31)	0.083
CFA diameter (per 1-mm decrease)	1.09 (0.95–1.25)	0.229	1.08 (0.95–1.22)	0.243
Puncture depth (per 1-mm decrease)	1.04 (1.00–1.08)	0.055	1.04 (1.00–1.09)	0.067

Surgical conversion, 39 of 485 limbs (see Methods for definition and model); exploratory analysis given the limited number of events.
CFA, common femoral artery; CI, confidence interval
